# Functional neuroimaging of the human lateral septum: a systematic review of behavioral correlates and anatomical localization

**DOI:** 10.1093/scan/nsag064

**Published:** 2026-08-08

**Authors:** Julie Klinke, Kaiwen Zhang, Nicole Li, Amir Alidousti, Filip Gedin, Valentina Molinari, William H Thompson, Saida Hadjab, Karin Jensen

**Affiliations:** Department of Clinical Neuroscience, Karolinska Institutet, Stockholm, 17176, Sweden; Department of Neuroscience, Karolinska Institutet, Stockholm, 17176, Sweden; Department of Neuroscience, Karolinska Institutet, Stockholm, 17176, Sweden; Department of Clinical Neuroscience, Karolinska Institutet, Stockholm, 17176, Sweden; Department of Clinical Neuroscience, Karolinska Institutet, Stockholm, 17176, Sweden; Department of Clinical Neuroscience, Karolinska Institutet, Stockholm, 17176, Sweden; Department of Clinical Neuroscience, Karolinska Institutet, Stockholm, 17176, Sweden; Department of Applied Information Technology, Gothenburg University, Gothenburg, 40530, Sweden; Department of Neuroscience, Karolinska Institutet, Stockholm, 17176, Sweden; Department of Clinical Neuroscience, Karolinska Institutet, Stockholm, 17176, Sweden

**Keywords:** lateral septum, social cognition, sexual arousal, cognition

## Abstract

The lateral septum (LS) is a basal forebrain structure extensively studied in rodents, where it regulates social behavior, stress, and motivation. In humans, its subtle anatomy and poor delineation limit systematic investigation with non-invasive imaging. We present the first systematic review of functional neuroimaging studies reporting LS activation in humans. Following PRISMA guidelines and a preregistered protocol, we found 3728 records. After screening, 26 studies met inclusion criteria. LS activations were categorized by behavioral domain, and spatial coordinates mapped for convergence. Reported LS/septal area activation was associated with prosocial processes including empathy, caregiving, trust, cooperation, and charitable decisions. LS activation was also associated with affiliative reward, sexual arousal, and affective states like guilt and gratitude, with alterations noted in depression. Coordinate-based mapping (where available) showed a tentative tendency toward left/anterior localization for social cognition-related activations in the left anterior septal area, though localization varied across studies due to anatomical and methodological challenges. Overall, the literature suggests the LS/septal area may contribute to multiple social-affective and motivational processes, but anatomical specificity remains uncertain. Despite challenges to measure LS function in humans, new neuroimaging techniques may elucidate LS function. This review highlights the LS as an underexplored but potentially critical structure for human socio-emotional and motivational processes, with clinical relevance for psychiatric conditions.

## Introduction

The lateral septum (LS) is a subcortical structure within the basal forebrain, situated in the septal area of the brain. The septal area consists of a collection of interconnected nuclei located near the midline of the brain, between the lateral ventricles. It is medially bordered by the medial septal nucleus and the septum pellucidum. The LS extends anteriorly toward the nucleus accumbens and the preoptic area, while posteriorly it transitions into the hypothalamus and the anterior commissure (Andy and Stephan 1968). It is recognized as a component of the limbic system, with strong interconnections to the hippocampus (Amaral and Cowan 1980), and is characterized by GABAergic input from the hippocampus (Gallagher *et al.* 1995), suggesting an inhibitory role in the modulation of neural circuits linked to both cognitive and affective processes (Menon *et al.* 2022).

Even if the LS is widely mentioned in the neuroscience literature, the majority of studies have been performed in rodents. Anatomically, the LS differs significantly between rodents and humans (Sheehan *et al.* 2004). In rodents, the LS is a proportionally large and well-defined structure with clearly distinguishable borders and subdivisions that are easy to identify in histological sections. It is commonly divided into subregions, each with distinct connectivity and functions (Andy and Stephan 1968). In humans, the LS is considerably smaller and less distinct, making it difficult to isolate from neighboring structures, even if attempts to outline the LS from high-resolution magnetic resonance imaging (MRI) have been demonstrated (Butler *et al.* 2014). Its internal organization is not well characterized, and standard neuroanatomical atlases for humans rarely include it as a separate structure (Fischl *et al.* 2002, Tzourio-Mazoyer *et al.* 2002). Another reason why the human LS may have been overlooked relates to the longstanding notion that the septal nuclei are vestigial in humans and not central for understanding human behavior (Andy and Stephan 1968). These anatomical and methodological differences have hindered the translation of findings from animal models to the human brain and created a major caveat in the understanding of LS function in humans. In rodents, the LS regulates social behavior (e.g. affiliation, aggression), stress responses, and motivational states (Menon *et al*. 2022, Sheehan *et al*. 2004). Given these well-documented functions and the LS’s evolutionary conservation, investigating whether similar or divergent roles exist in humans is theoretically important for understanding the neural basis of social-affective processing across species.

Functional neuroimaging is a non-invasive tool that allows for mapping of the functions of the human brain. Since functional MRI (fMRI) became widely available for researchers in the early 2000s, it has become the most widely used technique for functional neuroimaging in humans (Chen and Glover 2015). This is likely because fMRI is completely non-invasive (no ionizing radiation), allows for high spatial resolution (often between 2 and 4 mm), and provides coverage of the entire brain including deep subcortical structures. fMRI can thus be used to link the function of these structures to specific tasks, emotional states, and cognitive processes, and to explore group differences across populations in a non-invasive way.

Previous studies have attempted to outline the LS in humans using anatomical MRI images (Butler *et al.* 2014), and reviews have summarized the potential function of the LS based on rodent studies (Sheehan *et al.* 2004). However, a systematic investigation of the functional role of the LS in humans is lacking. Here, we performed a systematic review of all functional brain imaging studies in humans that report activations in the LS. The goal was to (i) outline the different categories of behavior associated with LS activations in humans and (ii) provide a heat map of the spatial coordinates reported as LS activations in these studies.

## Materials and methods

This study was conducted in accordance with the PRISMA guidelines. The study protocol was preregistered at Open Science Foundation (https://osf.io/3uypg). No changes were made after registration.

### Search strategy

The literature search was performed by a specialist at the Karolinska Institute University Library (KIB) using the following databases: Medline, Web of Science, and PsycInfo. All research studies that fulfilled the following inclusion criteria were included: (i) used functional brain imaging, (ii) were conducted in humans, and (iii) reported neural activations in the LS/septal area. We distinguish “LS-specific” studies with coordinates or anatomical description within LS boundaries from “septal area” studies that use a broader definition (e.g. “septal area,” “septo-hypothalamic region”; see Table 2). The last search was conducted on 8 February 2024. The search strategy was developed in Medline (Ovid) in collaboration with librarians at KIB. For each search concept, Medical Subject Headings terms and free text terms were identified. The search was then translated, in part using Polyglot Search Translator (Clark *et al.* 2020), into the other databases. Databases were searched from inception. Animal studies were excluded using available search blocks for Medline and PsycInfo and Web of Science. The strategies were peer reviewed by another librarian prior to execution. De-duplication was done using the method described by Bramer *et al*. (2016). One final, extra step was added to compare DOIs. The full search strategy syntax for all databases is available in the Supplementary Appendix.

Following the search, a total of 3728 titles and abstracts were identified and screened for eligibility by two independent reviewers using the Rayyan software for meta-analyses (Qatar Computing Research Institute), leading to the identification of 29 studies. Next, full-text articles were reviewed by the same independent reviewers. This phase further narrowed down the selection based on article accessibility (*n* = 1) and whether the studies explicitly reported coordinates for the LS or septal area, septal nuclei (*n* = 2). Only studies meeting both criteria were retained, corresponding to 26 studies. Any misalignment during screening was resolved through discussion with a third.

All data from the 26 resulting studies regarding sample size, study population characteristics, and brain imaging results (including brain coordinates) were extracted by two independent reviewers and compiled by a third reviewer (see Table 1). Methodological information, including scanner type and resolution, analysis software, voxel and smoothing kernel size, normalization atlas, and analysis approach that led to septum activation findings (a priori vs whole-brain) are available in Table 2.

**Table 1:** Overview and conclusions of the included studies.

Author, year	Category/type of behavior	Sample size (*n*)	Population	Mean age	Task during fMRI	Main conclusions
Bortolini *et al.* 2021	Reward (Social function)	23	Healthy	25.9	Affiliative Motive Incentive Delay (AMID) task: anticipation and viewing of affiliative and non-affiliative video rewards.	Affiliative reward outcomes selectively activated the septo-hypothalamic area, which correlated with positive arousal and re-exposure motivation.
Chen *et al.* 2020	Social function	304	Healthy	M: 20.7 F: 20.5	Prisoner’s Dilemma game: Participants engaged in repeated social interactions (reciprocated/unreciprocated cooperation) 40 minutes after intranasal oxytocin or placebo administration.	In rs53576 GG women, high oxytocin receptor methylation enhanced oxytocin-related septum activation during mutual cooperation, suggesting increased reward sensitivity to prosocial interactions under specific epigenetic conditions.
Diaconescu *et al.* 2017	Social learning (Social function)	82	Healthy	25	Social outcome-prediction task: Participants received valid or misleading advice from a partner to guide lottery choices.	Septum responded to social prediction errors and shifts in adviser trustworthiness, suggesting its role in uncertainty monitoring.
Green *et al.* 2012	Depression and guilt (Emotion)	47	MDD and healthy controls	Control: 22.8 MDD: 25.6	Participants read guilt and indignation-inducing sentences describing morally inappropriate actions by self or others; emotional response and neural activity were then measured.	MDD was associated with guilt-selective reduced ATL–septum coupling, indicating a link between overgeneralized self-blame and network dysfunction.
Green *et al.* 2013	Emotional integration (Emotion)	56	MDD and healthy controls	Control: 22.7 MDD: 25.6	Participants read social behavior scenarios varying in agency (self vs other) and valence, selected the best-fitting social concept among similar alternatives, and rated emotional unpleasantness.	Increased conceptual-emotional interdependence in MDD was linked to overgeneralized self-blame and reduced ATL–septum connectivity during guilt; strong ATL–septum coupling protected against maladaptive self-appraisal.
Hazlett *et al.* 2021	Emotional/gratitude (Emotion)	61	Healthy	43.1	Participants completed a gratitude-induction task and a threat-reactivity task while neural and inflammatory responses were recorded.	Caregiving-related septum activity during gratitude did not predict reduced threat response; only support-giving behavior linked to lower amygdala activity and inflammation.
Ho *et al.* 2014	Social function (Social function)	33	Healthy	20.06	Participants responded to a child’s needs (food or water) based on visual cues and received positive or negative child feedback; brain responses were examined in relation to empathy traits.	Among four empathy traits (Fantasy, Empathic Concern, Perspective Taking, Personal Distress), only Fantasy—reflecting imaginative identification with others—was linked to septum activation during negative feedback; greater septum–hypothalamus coupling predicted lower stress reactivity.
Hsu *et al.* 2008	Moral/decision making (Social function)	26	Healthy	39.2	Participants decided how to allocate monetary donations to orphaned children in Uganda during a charitable giving task.	The septal–subgenual region was involved in integrating social and moral values into decisions, supporting its role in social attachment, trust, and charitable giving.
Inagaki and Eisenberger 2012	Empathy for pain/social (Social function)	40	Healthy	Not stated	Female participants held either their male partner’s arm or a squeeze ball while anticipating their partner receiving a shock; control conditions included no shock or no contact.	Support giving (arm holding during partner’s pain) selectively activated the septum, suggesting its role in empathic caregiving and social buffering.
Inagaki et al. 2016	Social function	36	Healthy	22.36	Stress (based on the Montreal Imaging stress task), affiliative (viewing close others), and prosocial (support-giving) tasks.	Support giving (but not receiving) activated the septum, suggesting its role in social reward and stress regulation.
Inagaki and Ross 2018	Social function	427	Healthy	40.84	Task to give targeted (loved one), untargeted (unknown), or self-reward (raffle tickets), followed by an emotional faces task during fMRI.	Targeted support to a close other activated septum and reduced amygdala reactivity, indicating stress-buffering effect.
Kim and Jeong, 2014	Sexual arousal	24	Healthy	M: 23.5 F: 22.7	Participants viewed erotic video clips and rated sexual arousal after scanning.	Septum activation was stronger in males than females during sexual arousal, suggesting sex differences in emotional-motivational responses
Kim *et al.* 2009	Social compassion (Social function)	21	Healthy	28.1	Participants adopted either a compassionate or neutral mindset while viewing sad and neutral faces.	Compassionate attitude toward sad faces increased septum and ventral striatum activity, supporting their role in empathy-related emotion.
Kirk *et al.* 2016	Economic decision making and mindfulness (Social function)	51	Healthy	Control: 31.1 Mindfulness training: 32.2	Participants played the Ultimatum Game after mindfulness training or no training.	Mindfulness training increased cooperation and septum activation, suggesting enhanced prosocial decision-making.
Krueger *et al.* 2007	Trust/social function (Social function)	44	Healthy	28.3	Participants participated in a trust game with a stranger.	Unconditional trust selectively activated the septal area, indicating its role in social bonding.
Lorberbaum *et al.* 2002	Social function/maternal (Social function)	10	Healthy	30.55	First-time mothers listened to sounds of infants crying and white noise during an fMRI scan.	Infant crying activated the septum, suggesting its involvement in maternal affiliative response.
Lythe et al. 2015	Depression/self-blame (Emotion)	104	MDD patients in remission and healthy controls	Control: 33.4 MDD: 34.1	Participants read self- or other-blaming sentences and reported emotional reactions.	Greater temporal-septal connectivity was associated with self-blame processing in MDD, linking the septum to maladaptive emotion regulation.
Moll et al. 2012	Social function/affiliative emotion (Social function)	27	Healthy	27.3	Participants read and evaluated social scenarios with positive, neutral, or negative affiliative content.	Septum activation was elicited by affiliative content, suggesting a sensitivity to prosocial cues.
Morelli and Lieberman 2013	Social function/Empathy (Social function)	32	Healthy	19.9	Participants viewed emotional or neutral faces and were instructed to empathize, not empathize, or memorize.	Septum activation occurred across conditions, indicating a general role in emotional processing beyond explicit empathy.
Morelli *et al.* 2014	Social function/Empathy (Social function)	32	Healthy	19.9	Participants empathized with people experiencing pain, anxiety, or happiness depicted in photos.	Septum activity predicted individual empathy scores across emotional conditions, supporting its role in prosocial responsiveness.
Oh *et al.* 2012	Sexual arousal/transgender (Sexual arousal)	9	Healthy transgender individuals, post-surgery	39.7	Transgender participants viewed erotic nude images of males and females in alternating blocks.	Septum and hypothalamus activated only during female-nude viewing, suggesting involvement in sexual behavior.
Rilling *et al.* 2017	Social function/attractiveness (Social function)	80	Healthy/nasal spray	23.89	Participants viewed faces of same-/opposite-gender individuals or objects and rated approachability and attractiveness after intranasal arginine vasopressin or placebo at two time points.	Arginine vasopressin increased lateral septum and nucleus accumbens activity to female faces in men; left septum response persisted over time, suggesting involvement in affiliative processing.
Rilling *et al.* 2018	Social function	304	Healthy/nasal spray	M: 20.7 F: 20.5	Iterated Prisoner’s Dilemma game: participants engaged in repeated social interactions (reciprocated/unreciprocated cooperation) after intranasal oxytocin or placebo.	Oxytocin receptor methylation modulated oxytocin effects on brain response to social interactions, including in the lateral septum, precuneus, and visual cortex.
Yang *et al.* 2008	Sexual arousal	16	MDD/Healthy controls	HC: 40.3 Depression group: 41.7	Participants viewed erotic video clips after a period of abstinence; depressive and healthy women were compared.	Depressed females showed reduced septum activation during sexual arousal, suggesting dysfunction in motivational systems.
Yang *et al.* 2012	Sexual arousal	16	MDD/Healthy controls	HC: 40.3 Depression group: 41.7	Depressed women viewed erotic videos before and after antidepressant treatment (mirtazapine and as-needed benzodiazepine).	Treatment increased sexual arousal and septum, hypothalamus and parahippocampal gyrus activation, linking the activation of these areas to treatment-related improvements of sexual arousal in women with depression.
Yu *et al.* 2017	Social function	27	Healthy	22	Participants played a multi-round interactive game involving pain stimulation. During fMRI, they transferred money to a partner who bore part of their pain either intentionally or unintentionally. Intentional help led to lower experience of pain, higher reciprocity, and increased interpersonal closeness toward the partner.	The septum selectively encoded intentional help (gratitude) vs unintentional help, supporting its role in social reward processing.

**Table 2:** Methodological approaches of the included studies.

Author, year	Scanner quality	Analysis software	Smoothing kernel (mm)	Voxel size (mm)	Atlas used for normalization	A priori vs whole-brain analysis	LS vs SA	Included in Fig. 1; [coordinates]
Bortolini *et al.* 2021	3T Prisma scanner (Siemens)	SPM12	4	2 × 2 × 2	MNI152NLin2009cAsym	A priori	SA (septo-hypothalamic region)	No
Chen *et al.* 2020	3 T Siemens Trio	FSL	5	1 × 1 × 1	MNI	Whole brain	LS	Yes; [−4, 12, 6]
Diaconescu et al. 2017	3 T Philips Achieva; 3 T Philips Ingenia	SPM12	6	2 × 2 × 3	MNI	Whole brain + a priori	Medial and lateral areas of the LS were part of a combined ROI covering the basal forebrain; septal activation was reported on in isolation.	Yes; [−5, 10, -7]
Green *et al.* 2012	3T Philips Achieva	SPM8	6	2.29 × 2.29 × 3 mm (reconstructed)	SPM8 standard T1 template	A priori	SA (subgenual cingulate cortex and adjacent septal region)	Yes; [−6, 22, 0]
Green *et al*. 2013	3 T Philips Achieva	SPM8	6	2.29 × 2.29 × 3 mm (reconstructed)	SPM8 standard T1 template	A priori	SA (reports on the septal area of the nucleus accumbens, and the subgenual cingulate/septal area)	Yes; [8, 6, 4]
Hazlett *et al.* 2021	3 T Siemens Trio	SPM12	8	?	MNI	A priori	SA	Yes; [0, 2, −4]
Ho *et al.* 2014	3 T Philips	SPM8	8	3.44 × 3.44 × 2.80; normalized functional images were resliced to 2 × 2 × 2	SPM8 DARTEL method	A priori	SA	Yes; [−4, 4, 6]
Hsu *et al.* 2008	3T Siemens Trio	SPM2	8	1 × 1 × 1	MNI	?	SA (caudate/septal–subgenual region)	Yes; [12, 21, 3]
Inagaki and Eisenberger 2012	3 T Siemens Trio	SPM5	8	1.6 × 1.6 × 3	Standard stereotactic space	A priori	SA	Yes; [0, 2, −4]
Inagaki *et al.* 2016	3 T Siemens Tim Trio	SPM8	8	?	MNI	A priori	SA	No
Inagaki and Ross, 2018	Study I: 3 T Siemens Magnetom Allegra Study II: 3 T Siemens Trio TM	SPM8	Study I: 8 Study II: 6	?	MNI	A priori	SA	No
Kim and Jeong 2014	3.0 Tesla Forte (Isol Technology)	SPM2; FALBA	8	?	MNI	A priori	SA	Yes; [−1, 7, −9]
Kim *et al.* 2009	3 T Philips	SPM2	7	1.80 × 1.80 × 4	SPM2 MNI T1 template	A priori	SA (ventral striatum/septal region)	Yes; [7, 12, −5] and [−7, 12, −5]
Kirk *et al.* 2016	3T Siemens Trio	SPM8	8	3.4 × 3.4 × 4	SPM8 MNI template	A priori	SA	Yes; [−4, 4, 6]
Krueger *et al.* 2007	3 T GE Medical Systems	BrainVoyager QX	8	3.75 × 3.75 × 6	Talairach	A priori	SA	Yes; [6, 11, 4]
Lorberbaum *et al.* 2002	1.5 T Picker Edge	SPM96	6	3 × 3 × 3 (after normalization)	Talairach	Whole-brain	SA	Yes; [6, 6, 6]
Lythe *et al.* 2015	3 T Philips Achieva	SPM8	6	2.29 × 2.29 × 3 (reconstructed)	SPM8 template	A priori	SA (subgenual cingulate cortex and adjacent septal region)	Yes; [2, 14, −6]
Moll *et al.* 2012	3 T Philips Achieva	SPM8	8	2 × 2 × 2	MNI	A priori	SA (basal forebrain ROI encompassing the septal/preoptic-anterior hypothalamic structures)	No
Morelli and Lieberman 2013	3 T Siemens Trio	SPM8	8	3 × 3 × 3 (resliced)	MNI standard stereotactic space using scalped ICBM152 template	A priori	SA	No
Morelli *et al.* 2014	3 T Siemens Trio	SPM8	8	3 × 3 × 3 (resliced)	MNI standard stereotactic space using scalped ICBM152 template	A priori	SA	No
Oh *et al.* 2012	3T Siemens Magnetom	SPM2	8	?	MNI	A priori	SA	No
Rilling *et al.* 2017	Not stated	FSL	4	?	MNI	Whole brain + a priori	LS	No
Rilling *et al.* 2018	3 T Siemens Trio	FSL	5	1 × 1 × 1	MNI	A priori	LS	No
Yang *et al.* 2008	1.5T, Sigma Horizon, GE Medical Systems	SPM99	8	?	MRIcrow1.36 and SPM99 used to align images to template brain approximating Talairach	Whole brain	SA	No
Yang *et al.* 2012	1.5T GE Medical Systems	SPM2	8	?	MRIcrow1.36 and SPM99 used to align images to template brain approximating Talairach	Whole brain	SA	No
Yu *et al.* 2017	3T Siemens Trio	FSL	8	1 × 1 × 1 at data collection; reassembled to 3 × 3 × 3 during preprocessing	MNI	Whole brain + a priori	SA	No

ROI, region of interest; SA, septal area.

**Figure 1:**
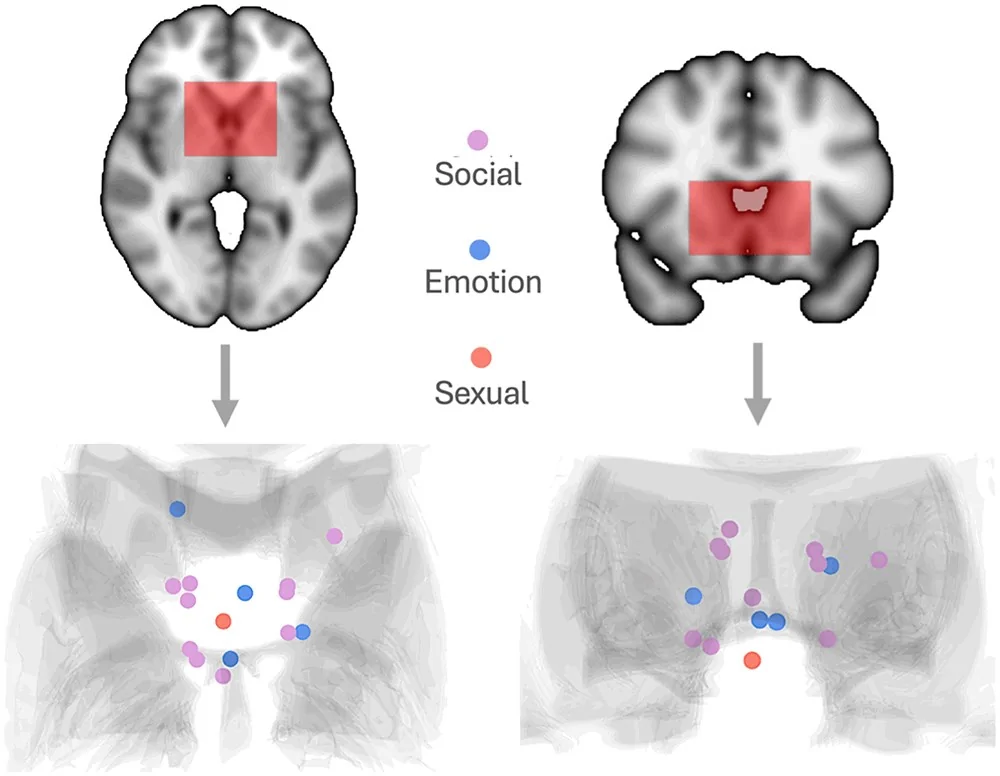
Descriptive plot of reported LS activation coordinates (*k* = 14) in MNI space, color-coded by behavioral domain (social, emotional, sexual). Social cognition-related activations showed a preliminary tendency toward left/anterior localization in the septal area. No clear patterns emerged for emotional or sexual domains. Only studies reporting precise coordinates for LS ROI or peak activations were included. The visualization is intended as hypothesis generating and provided for illustrative purposes only. Please see Table 2 for an overview of the included studies.

The extracted brain coordinates were used to generate a descriptive visualization of septal activation across studies after converting all coordinates to the same Montreal Neurological Institute (MNI) space (Fig. 1). Only studies with precise extractable coordinates (*k *= 14) contributed to the descriptive Fig. 1; if coordinates were reported as a range or an anatomical area, they were not included in the visualization. For one study, two peaks/contrasts were available; both are included in Fig. 1. All 26 studies contributed to the thematic synthesis regardless of exact coordinate availability. For an overview of which studies are included in Fig. 1, see Table 2.

## Results

### Overview

A total of 26 studies fulfilled the inclusion criteria for the present systematic review. Of these, only two studies explicitly reported results from the LS, while the remaining 24 reported septal area activation. Fourteen studies provided precise brain coordinates that could be used for the descriptive Fig. 1 (also see Table 2). All contributed to the thematic synthesis. The studies had on average 74 participants, and a majority of the studies (*k *= 20) were performed in healthy populations (see Table 1). The mean age of the participants was 28.7 years. The studies were published between year 2002 and 2021 and originated from six countries. A summary of the individual studies’ findings can be found in Table 1.

### Specific functions

#### Social cognition

According to this systematic review, the LS appears to be involved in various social functions in humans, such as empathy, trust evaluation, cooperation, affiliative responses, and social reward. Its activation across different pro-social scenarios suggests a role in processing and encoding social cues and in selecting appropriate behavioral responses.

Fairness and cooperation, two fundamental components of human social interactions, have also been linked to LS activity. Hsu *et al.* (2008) asked participants to perform a monetary distribution task designed to contrast equity with efficiency. Their results showed that the septal region was activated when participants made decisions of high utility, a composite measure integrating both efficiency and inequity. This finding, in a relatively small group of participants (*n* = 26), suggests that the septal region functions as a hub for integrating multiple social values during decision-making. Later, another study (Kirk *et al.* 2016) extended this work by studying the effects of mindfulness training on cooperative behavior in the ultimatum game. They found that septal region activity was associated with a greater tendency to accept unfair monetary offers following mindfulness training, suggesting that prosocial behavior shaped by training interventions is encoded in this region.

While these studies show intriguing links between mindfulness, cooperation, and septal region activity, the small sample and limitations of using experimental games to illustrate real-world social behavior mean that the data should be interpreted carefully and replicated in larger, more diverse cohorts.

Further evidence of the social role of the LS comes from Rilling *et al.* (2017), who investigated the effects of intranasal arginine vasopressin (AVP) administration on neural responses to faces. Even if AVP had no effect on subjective judgments of the other sex, the results indicate that AVP increased LS activation in males viewing female faces (but not vice versa), both immediately and up to 2 weeks later. Although the study was based on watching photos and not a naturalistic social setting, it indicates sex-specific activations of the LS in response to AVP, with implications for social and potentially romantic partner processing.

#### Reward

In addition to its role in social cognition, the LS has been implicated in reward processing, particularly in affiliative contexts. In a study by Bortolini *et al.* (2021), the authors employed video clips of affiliative and non-affiliative behaviors to examine the neural correlates of reward anticipation and outcome. They reported activation of the septo-hypothalamic area, including the LS, specifically in response to affiliative rewards in the form of visual stimuli evoking emotions such as eroticism, tenderness, and awe. Importantly, activity in this region during the outcome phase correlated with participants’ self-reported positive arousal and motivation to re-watch reward videos. Although the sample size was small (*n* = 23), the study provides preliminary evidence for LS involvement in affiliative reward processing. The use of videos to depict affiliative and non-affiliative rewards may not fully capture the complexity of real-life social interactions and emotions. However, the study provides a foundation for future studies exploring the role of the LS in human motivation and reinforcement.

#### Emotions

Some evidence indicates that the LS, particularly through its connectivity with the anterior temporal lobe (ATL), contributes specifically to the processing of guilt. Green *et al.* (2012) found that in patients suffering from major depressive disorder (MDD), reduced ATL–LS coupling was associated with negative and maladaptive self-related processes. The LS also appears to be involved in socially directed emotions such as gratitude, specifically in distinguishing voluntary (intentional) help from involuntary help (Yu *et al.* 2017). As in many other experiments, it is unclear how a computerized experimental task of helping behavior may generalize to real-world behaviors.

#### Sexual behavior

There are indications of sex differences in LS activations during erotic stimuli. Kim and Jeong (2014) observed greater LS activation in males compared to females when viewing erotic video clips, indicating a sex-specific response in sexual arousal processing. A small study by Oh *et al.* (2012) (*n* = 9) reported that brain activation patterns in transgender women differed when viewing male versus female nude images. Specifically, viewing female nude images predominantly activated the hypothalamus and septal area, despite the participants’ stated exclusive attraction to males. These findings suggest that the septal area is involved in processing sexual stimuli and, potentially, in sexual behavior.

Additionally, the LS has been implicated in the neurobiology of sexual dysfunction associated with MDD. Yang *et al.* (2008) found lower LS activation in women with MDD and comorbid sexual arousal disorder compared to healthy controls when presented with erotic video stimulation. In a follow-up study done on the same cohort, Yang *et al*. (2012) demonstrated that participants showed normalized LS activation in response to erotic video stimuli following treatment with the antidepressant medication mirtazapine. This small study, with less than 10 individuals in each group (and no placebo control), provides an indication that LS activation in women may be related to sexual reactivity.

In summary, neuroimaging studies have established the LS as an important mediator of both physiological and dysfunctional sexual arousal in humans, with potential implications for understanding and treating sexual arousal disorders.

## Discussion

This systematic review provides the first comprehensive synthesis of human functional neuroimaging studies that report activation in the LS. Despite its limited anatomical volume and poor delineation (Butler *et al.* 2014), as well as underrepresentation in standard neuroimaging parcellations of the human brain (Fischl *et al.* 2002, Tzourio-Mazoyer *et al.* 2002), LS/septal area activation was reported across diverse tasks, involving social cognition, emotional regulation, sexual behavior, and moral decision-making, though anatomical specificity is limited by fMRI resolution and heterogeneous labeling. These results parallel the well-documented functions of the LS in rodent studies, where it has been shown to play a role in social behaviors, stress responses, and motivational states (Sheehan *et al.* 2004). These behavioral functions are thought to be exerted through the LS interconnections with other limbic and hypothalamic structures, e.g. the hippocampus.

One of the most striking patterns observed across studies was the frequent involvement of the LS/septal area in paradigms requiring prosocial behavior, caregiving, empathy, and trust. Tasks that involved giving support to others, engaging in cooperative decision-making, or responding to emotionally salient social cues (e.g. infant cries or facial expressions) reliably engaged the LS/septal area. These findings align with hypotheses derived from rodent research suggesting a role of the LS in affiliative and prosocial processes, including social attachment. However, such interpretations should be made cautiously, as reverse inference remains a significant limitation. Specifically, activation of the LS during prosocial paradigms in humans does not necessarily indicate that the region selectively encodes prosociality or attachment per se, but may instead reflect broader processes recruited across these tasks, such as salience detection, affective arousal, motivational significance, social relevance, or autonomic regulation. Similarly, several studies reported LS activation during moral decision-making or charitable giving, suggesting a role in integrating affective and social information into value-based judgments. This aligns with earlier views of the LS/septal area as a modulatory hub that balances affective arousal with social context, likely via its reciprocal connections with the hypothalamus, amygdala, and prefrontal regions.

The LS also appears to be sensitive to sexual and emotional arousal, though the number of studies investigating these domains was more limited. Activation patterns suggest that the LS may contribute to the processing of motivational salience in sexual stimuli, with differences observed across sex and clinical populations. For example, patients with unmanaged MDD showed altered LS connectivity, especially in the context of increased self-blame and reduced affiliative motivation, which may reflect broader disruptions in limbic network function. Conversely, the same cohort exhibited treatment-induced increased LS activation, which was associated with increased sexual motivation. Interestingly, in male-to-female transgender participants with a stated exclusive attraction to men, LS activation was seen only when participants were exposed to visual stimuli of female nudity. This could potentially indicate that LS activation, while seemingly related to sexual behavior or processing of sexual stimuli, may not necessarily be linked to sexual arousal per se.

Despite these promising results, several methodological challenges should be acknowledged. First, this systematic review is inherently limited to studies that explicitly reported activations within the LS or septal area. Consequently, the review cannot determine how often this area was examined but not reported, either because no significant effects were observed, the region was not analyzed in sufficient detail, or findings fell below statistical or reporting thresholds. As a result, the present synthesis may be influenced by reporting bias and should not be interpreted as an estimate of the overall frequency or selectivity of LS involvement across neuroimaging paradigms.

The LS in humans is a small and anatomically indistinct structure, often measuring less than 4 mm in width (Butler *et al.* 2014). This makes it difficult to localize with certainty using standard fMRI techniques, particularly when spatial resolution is coarse or smoothing kernels are large (often between 4 and 8 mm). Also, the cluster statistics often employed in fMRI analyses make it difficult to know the exact location of a significant activation that often encompasses a volume greater than the LS. As per Fig. 1, our review highlights a tendency toward anatomical liberty in the definition of the LS across studies. Some studies provided explicit foci in the form of region of interest (ROI) center coordinates or peak activation coordinates in standardized MNI or Talaraich space, while others reported activations of the LS that overlapped with other parts of the septal area or even the hypothalamus and nucleus accumbens. Others again referred to the “septal area” or “septohypothalamic region” without providing precise anatomical definitions or referencing consistent coordinate-based criteria. Whereas animal research makes use of clearer segmentation (post mortem) within the LS and related projection and function (Sheehan *et al.* 2004), in human fMRI research, it remains difficult to determine whether the same subregion(s) of the LS are consistently engaged across different functions, or whether partially distinct LS subdivisions mediate different behavioral domains. As such, the inconsistencies seen in the cited literature here may have been caused or corroborated by the use of smoothing kernels of up to 8 mm, different spatial resolutions, or the use of anatomical atlases with no standardized LS parcellation. This variance in approaches poses issues for cross-study comparisons and complicates efforts to build cumulative knowledge about LS function in humans.

These challenges posed limitations to our ability to visualize findings in Fig. 1. Since precise coordinates were only available in 14 studies, the visualization cannot and should not be used for the interpretation of spatial convergence. Rather, the visualization should be treated as hypothesis generating only and will require replication from future studies with higher-field MRI (≥7 T), smaller voxels (≤2mm), minimal smoothing, and predefined LS ROIs based on high-resolution anatomy.

A final limitation comes from the lack of a formal quality assessment of the included studies. Unfortunately, we were unable to identify an established assessment tool that would be appropriate for the evaluation of observational human neuroimaging studies and were forced to rely on internal evaluation of methodological detail to estimate article quality. The development of such tools would be of great benefit for future systematic reviews and meta-analyses of human neuroimaging literature.

Despite these challenges, the present review underscores the feasibility and value of studying the LS with current neuroimaging techniques. For a long time, many studies may have only been able to identify LS activity using a priori ROIs. While this approach is beneficial from a statistical perspective, it illustrates how difficult it has historically been to investigate LS activation and function “organically,” i.e. exploratorily, in humans due to the diminutive nature of the region. With growing availability of high-field MRI (e.g. 7 T scanners), finer-resolution fMRI, and improved anatomical atlases, the neuroimaging field may be helpful in better delineating the LS in the human brain.

## Conclusion

The involvement of the LS/septal area has been reported across social cognition, reward, emotion, and sexual behavior, suggesting multifunctional contributions. Rather than serving a single isolated function, the LS/septal area may integrate diverse streams of social-affective and motivational information to shape adaptive behavioral responses. Its involvement in fairness and cooperation supports its role in value-based decision-making; its engagement in affiliative reward and sexual stimuli indicates an involvement in motivational processing and sexual behavior; and its modulation in affective disorders points toward potential clinical significance. The preliminary evidence suggests that the LS is not only central to understanding normative social and emotional behavior but may also be a potential target for investigating dysfunctions in sociality, motivation, and sexual function. In contrast to the notion of septal nuclei as vestiges in human brain function, this review highlights the importance of including small, evolutionarily conserved brain structures in the study of human behavior and emotion. Future studies with high-field MRI, LS-specific ROIs, and preprocessing pipelines intended for the study of exceedingly small anatomical locations will help better understand its role in human behavior.

## Supplementary Material

nsag064_Supplementary_Data

## Data Availability

The data from the current study will be available from the corresponding authors on request.

## References

[nsag064-B1] Amaral DG , Cowan WM. Subcortical afferents to the hippocampal formation in the monkey. J Comp Neurol 1980;189:573–91. 10.1002/cne.901890402 6769979

[nsag064-B2] Andy OJ , Stephan H. The septum in the human brain. J Comp Neurol 1968;133:383–410. 10.1002/cne.901330308 5710964

[nsag064-B3] Bortolini T , Melo B, Basilio R et al Striatal and septo-hypothalamic responses to anticipation and outcome of affiliative rewards. Neuroimage 2021;243:118474. 10.1016/j.neuroimage.2021.118474 34407439

[nsag064-B4] Bramer WM , Giustini D, de Jonge GB et al De-duplication of database search results for systematic reviews in EndNote. J Med Libr Assoc 2016;104:240–3. 10.3163/1536s050.104.3.014 PMC4915647 27366130

[nsag064-B5] Butler T , Zaborszky L, Pirraglia E et al Comparison of human septal nuclei MRI measurements using automated segmentation and a new manual protocol based on histology. Neuroimage 2014;97:245–51. 10.1016/j.neuroimage.2014.04.026 PMC4180657 24736183

[nsag064-B6] Chen JE , Glover GH. Functional magnetic resonance imaging methods. Neuropsychol Rev 2015;25:289–313. 10.1007/s11065-015-9294-9 PMC4565730 26248581

[nsag064-B229337829] Chen XU, , Nishitani S, , Haroon E et al Oxtr methylation modulates exogenous oxytocin effects on human brain activity during social interaction. Genes Brain and Behavior 2020;19. 10.1111/gbb.12555 30624029

[nsag064-B91250152] Clark JM, , Sanders S, , Carter M et al Improving the translation of search strategies using the Polyglot Search Translator: a randomized controlled trial. jmla 2020;108. 10.5195/jmla.2020.834 PMC7069833 32256231

[nsag064-B117496] Diaconescu AO, , Mathys C, , Weber LAE et al Hierarchical prediction errors in midbrain and septum during social learning. Soc Cogn Affect Neurosci 2017;12:618–34. 10.1093/scan/nsw171 PMC5390746 28119508

[nsag064-B7] Fischl B , Salat DH, Busa E et al Whole brain segmentation: automated labeling of neuroanatomical structures in the human brain. Neuron 2002;33:341–55. 10.1016/s0896-6273(02)00569-x 11832223

[nsag064-B8] Gallagher JP , Zheng F, Hasuo H et al Activities of neurons within the rat dorsolateral septal nucleus (DLSN). Prog Neurobiol 1995;45:373–95. 10.1016/0301-0082(95)98600-a 7617889

[nsag064-B9] Green S , Lambon Ralph MA, Moll J et al Guilt-selective functional disconnection of anterior temporal and subgenual cortices in major depressive disorder. Arch Gen Psychiatry 2012;69:1014–21. 10.1001/archgenpsychiatry.2012.135 22638494

[nsag064-B9820108] Green S, , Lambon Ralph MA, , Moll J et al The neural basis of conceptual–emotional integration and its role in Major Depressive Disorder. Social Neuroscience 2013;8:417–33. 10.1080/17470919.2013.810171 PMC3783899 23826933

[nsag064-B0328890] Hazlett LI, , Moieni M, , Irwin MR et al Exploring neural mechanisms of the health benefits of gratitude in women: a randomized controlled trial. Brain, Behavior, and Immunity 2021;95:444–53. 10.1016/j.bbi.2021.04.019 33932527

[nsag064-B40871324] Ho SS, , Konrath S, , Brown S et al Empathy and stress related neural responses in maternal decision making. Front Neurosci 2014;8. 10.3389/fnins.2014.00152 PMC4053926 24971049

[nsag064-B10] Hsu M , Anen C, Quartz SR. The right and the good: distributive justice and neural encoding of equity and efficiency. Science 2008;320:1092–5. 10.1126/science.1153651 18467558

[nsag064-B525315] Inagaki TK, , Bryne Haltom KE, , Suzuki S et al The neurobiology of giving versus receiving support. Psychosom Med 2016;78:443–53. 10.1097/PSY.0000000000000302 PMC4851591 26867078

[nsag064-B2699983] Inagaki TK, , Eisenberger NI. Neural correlates of giving support to a loved one. Psychosom Med 2012;74:3–7. 10.1097/PSY.0b013e3182359335 22071630

[nsag064-B4650483] Inagaki TK, , Ross LP. Neural correlates of giving social support: differences between giving targeted versus untargeted support. Psychosom Med 2018;80:724–32. 10.1097/PSY.0000000000000623 30148747

[nsag064-B8057232] Kim G-W, , Jeong G-W. A comparative study of brain activation patterns associated with sexual arousal between males and females using 3.0‑T functional magnetic resonance imaging. Sex Health 2014;11:11–6. 10.1071/SH13127 24331548

[nsag064-B1585121] Kim J-W, , Kim S-E, , Kim J-J et al Compassionate attitude towards others' suffering activates the mesolimbic neural system. Neuropsychologia 2009;47:2073–81. 10.1016/j.neuropsychologia.2009.03.017 19428038 19428038

[nsag064-B11] Kirk U , Gu X, Sharp C et al Mindfulness training increases cooperative decision making in economic exchanges: evidence from fMRI. Neuroimage 2016;138:274–83. 10.1016/j.neuroimage.2016.05.075 PMC4954868 27266443

[nsag064-B5031932] Krueger F, , McCabe K, , Moll J et al Neural correlates of trust. Proc Natl Acad Sci USA 2007;104:20084–9. 10.1073/pnas.0710103104 PMC2148426 18056800

[nsag064-B5992506] Lorberbaum JP, , Newman JD, , Horwitz AR et al A potential role for thalamocingulate circuitry in human maternal behavior. Biological Psychiatry 2002;51:431–45. 10.1016/S0006-3223(01)01284-7 11922877

[nsag064-B17291278] Lythe KE, , Moll J, , Gethin JA et al Self-blame–selective hyperconnectivity between Anterior Temporal and Subgenual Cortices and prediction of recurrent depressive episodes. JAMA Psychiatry 2015;72:1119. 10.1001/jamapsychiatry.2015.1813 26445229

[nsag064-B12] Menon R , Süß T, Oliveira VEdM et al Neurobiology of the lateral septum: regulation of social behavior. Trends Neurosci 2022;45:27–40. 10.1016/j.tins.2021.10.010 34810019

[nsag064-B03218796] Moll J, , Bado P, , de Oliveira-Souza R et al A neural signature of affiliative emotion in the human septohypothalamic area. J Neurosci 2012;32:12499–505. 10.1523/JNEUROSCI.6508-11.2012 PMC6621241 22956840

[nsag064-B4596372] Morelli SA, , Lieberman MD. The role of automaticity and attention in neural processes underlying empathy for happiness, sadness, and anxiety. Front Hum Neurosci 2013;7. 10.3389/fnhum.2013.00160 PMC3647144 23658538

[nsag064-B01347388] Morelli SA, , Rameson LT, , Lieberman MD. The neural components of empathy: predicting daily prosocial behavior. Soc Cogn Affect Neurosci 2014;9:39–47. 10.1093/scan/nss088 PMC3871722 22887480

[nsag064-B13] Oh SK , Kim GW, Yang JC et al Brain activation in response to visually evoked sexual arousal in male-to-female transsexuals: 3.0 tesla functional magnetic resonance imaging. Korean J Radiol 2012;13:257–64. 10.3348/kjr.2012.13.3.257 PMC3337861 22563262

[nsag064-B1747134] Rilling JK, , Chen X, , Chen XU et al Intranasal oxytocin modulates neural functional connectivity during human social interaction. American J Primatol 2018;80. 10.1002/ajp.22740 PMC6086772 29427292

[nsag064-B14] Rilling JK , Li T, Chen X et al Arginine vasopressin effects on subjective judgments and neural responses to same and other-sex faces in men and women. Front Endocrinol (Lausanne) 2017;8:200. 10.3389/fendo.2017.00200 PMC5566575 28871239

[nsag064-B15] Sheehan TP , Chambers RA, Russell DS. Regulation of affect by the lateral septum: implications for neuropsychiatry. Brain Res Brain Res Rev 2004;46:71–117. 10.1016/j.brainresrev.2004.04.009 15297155

[nsag064-B16] Tzourio-Mazoyer N , Landeau B, Papathanassiou D et al Automated anatomical labeling of activations in SPM using a macroscopic anatomical parcellation of the MNI MRI single-subject brain. Neuroimage 2002;15:273–89. 10.1006/nimg.2001.0978 11771995

[nsag064-B17] Yang JC , Park JI, Kim GW et al Effects of antidepressant treatment on sexual arousal in depressed women: a preliminary FMRI study. Psychiatry Investig 2012;9:379–83. 10.4306/pi.2012.9.4.379 PMC3521115 23251203

[nsag064-B18] Yang JC , Park K, Eun SJ et al Assessment of cerebrocortical areas associated with sexual arousal in depressive women using functional MR imaging. J Sex Med 2008;5:602–9. 10.1111/j.1743-6109.2007.00737.x 18194182

[nsag064-B19] Yu H , Cai Q, Shen B et al Neural substrates and social consequences of interpersonal gratitude: intention matters. Emotion 2017;17:589–601. 10.1037/emo0000258 27936814

