# Supplementary material for: Functional neuroimaging of the human lateral septum: a systematic review of behavioral correlates and anatomical localization
Source: Soc Cogn Affect Neurosci. 2026 Aug 8;21(1):nsag064. doi: 10.1093/scan/nsag064 (PMC13537321; doi:10.1093/scan/nsag064)
Supplement: Supplementary file 1 [file nsag064_supplementary_data.docx]

Documentation of search strategies

University Library search consultation group

Date: February 2024

Databases:

1. Medline (Ovid)
2. Web of Science Core Collection (Clarivate)
3. PsycInfo (Ebsco)

Total number of hits:

- Before deduplication: 5801
- After deduplication: 3728

**Identification of studies via databases and registers**

Records removed *before screening*:

Duplicate records removed (n = 2073)

Records marked as ineligible by automation tools (n = 0)

Records removed for other reasons (n = 0)

Records identified from:

**Databases (n = 5801)**

Medline (n = 2463)

Web of Science (n = 2241)

PsycInfo (n = 1097)

Registers (n = 0)

Records screened

(n = 3728)

Records excluded

(n = 3699)

Reports sought for retrieval

(n = 29)

Reports not retrieved

(n = 0)

**Screening**

Reports assessed for eligibility

(n = 29)

Reports excluded:

Wrong brain area (n = 2)

Full-length not found (n =1)

**Identification**

Studies included in review

(n = 26)

**Included**

1. Medline

| Interface: Ovid MEDLINE(R) ALL  Date of Search: 2024-02-08  Number of hits: 2463  Comment: In Ovid, two or more words are automatically searched as phrases; i.e. no quotation marks are needed | Field labels   - exp/ = exploded MeSH term - / = non exploded MeSH term - .ti,ab,kf. = title, abstract and author keywords - adjx = within x words, regardless of order - * = truncation of word for alternate endings |
| --- | --- |
| Ovid MEDLINE(R) ALL <1946 to February 07, 2024> 1 \| exp Brain/dg \| 105762; 2 \| exp Functional Neuroimaging/ \| 109266; 3 \| exp Magnetic Resonance Imaging/ \| 543310; 4 \| Magnetoencephalography/ \| 9461; 5 \| Neuroimaging/ \| 20164; 6 \| exp Positron-Emission Tomography/ \| 81516; 7 \| Nerve Net/ \| 34432; 8 \| ((chemical shift* or functional or magnetic resonance or MR or magneti#ation transfer* or positron-emission* or proton spin* or spin echo*) and (image or images or imaging or scan or scans or tomograp*)).ti,ab,kf. \| 569600; 9 \| (echoplanar* or echo-planar* or connectom* or functional cerebral localization* or fMRI or MRI or PET-MRI or PET or NMR or magnetoencephalogra* or magneto-encephalogra* or magneto encephalogra* or MEG or neuroimag* or zeugmatograph*).ti,ab,kf. \| 766360; 10 \| ((brain* or neur*) adj3 (activ* or connectiv* or correlate* or function* or image or images or imaging* scan or scans or mapping* or network* or tomograph*)).ti,ab,kf. \| 525715; 11 \| or/1-10 \| 1695678; 12 \| exp Septum of Brain/ \| 7220; 13 \| Paraterminal Bod*.ti,ab,kf. \| 3; 14 \| ((area or brain or nucle* or lateral* or lucidum* or pelusidum* or region) adj2 (anterior commissure or diagonal band or septal* or septum* or stria* or supracommissural or terminali*)).ti,ab,kf. \| 14293; 15 \| or/12-14 \| 19420; 16 \| 11 and 15 \| 5691; 17 \| (animals not humans).sh. \| 5159730; 18 \| 16 not 17 \| 2463 | |

2. Web of Science Core Collection

| Interface: Clarivate Analytics  Editions = A&HCI , ESCI , SCI-EXPANDED , SSCI  Date of Search: 2024-02-08  Number of hits: 2241 | Field labels   - TS/Topic = title, abstract, author keywords and Keywords Plus - NEAR/x = within x words, regardless of order - * = truncation of word for alternate endings   Note: the *Exact search*-function was used for all the searches |
| --- | --- |
| # \| Search Query \| Results; 1 \| (TI=(("chemical shift*" OR functional OR "magnetic resonance" OR MR OR "magneti?ation transfer*" OR positron-emission* OR "proton spin*" OR "spin echo*" ) AND (image OR images OR imaging OR scan OR scans OR tomograp* )) OR AB=(("chemical shift*" OR functional OR "magnetic resonance" OR MR OR "magneti?ation transfer*" OR positron-emission* OR "proton spin*" OR "spin echo*" ) AND (image OR images OR imaging OR scan OR scans OR tomograp*))) \| 541890; 2 \| (TI=(echoplanar* OR echo-planar* OR connectom* OR "functional cerebral localization*" OR fMRI OR MRI OR PET-MRI OR PET OR NMR OR magnetoencephalogra* OR magneto-encephalogra* OR "magneto encephalogra*" OR MEG OR neuroimag* OR zeugmatograph* ) OR AB=(echoplanar* OR echo-planar* OR connectom* OR "functional cerebral localization*" OR fMRI OR MRI OR PET-MRI OR PET OR NMR OR magnetoencephalogra* OR magneto-encephalogra* OR "magneto encephalogra*" OR MEG OR neuroimag* OR zeugmatograph*)) \| 1096739; 3 \| (TI=((brain* OR neur*) NEAR/2 (activ* OR connectiv* OR correlate* OR function* OR image OR images OR imaging* OR mapping* OR network*)) OR AB=((brain* OR neur*) NEAR/2 (activ* OR connectiv* OR correlate* OR function* OR image OR images OR imaging* OR mapping* OR network*))) \| 795286; 4 \| #1 OR #2 OR #3 \| 2067369; 5 \| (TI="Paraterminal Bod*" OR AB="Paraterminal Bod*") \| 3; 6 \| (TI=((area OR brain OR nucle* OR lateral* OR lucidum* OR pelusidum* OR region) NEAR/2 ("anterior commissure" OR "diagonal band" OR septal* OR septum* OR stria* OR supracommissural OR terminali* )) OR AB=((area OR brain OR nucle* OR lateral* OR lucidum* OR pelusidum* OR region ) NEAR/2 ("anterior commissure" OR "diagonal band" OR septal* OR septum* OR stria* OR supracommissural OR terminali* ))) \| 17784; 7 \| #5 OR #6 \| 17785; 8 \| #4 AND #7 \| 4579; 9 \| TS=((animal* OR rat OR rats OR mouse OR mice OR murine OR dog OR dogs OR canine OR cat OR cats OR feline OR rabbit OR cow OR cows OR bovine OR rodent* OR sheep OR ovine OR pig OR swine OR porcine OR veterinar* OR chick* OR zebrafish* OR baboon* OR nonhuman* OR primate* OR cattle* OR goose OR geese OR duck OR macaque* OR avian* OR bird* OR fish*) NOT (human* OR patient* OR women OR woman OR men OR man)) \| 5325132; 10 \| #8 NOT #9 \| 2241 | |

3. Psycinfo

| Interface: EBSCO  Date of Search: 2024-02-08  Number of hits: 1097 | Field labels   - DE = subject heading - TI = title - AB = abstract - KW = author keywords - Nx = within x words, regardless of order - * = truncation of word for alternate endings   Note: sometimes “quotation marks” are needed for single search terms to avoid automatic term mapping (lemmatization) |
| --- | --- |
| # \| Query \| Results; S13 \| S11 NOT S12 \| 1,097; S12 \| (PO Animal NOT PO Human) \| 390,290; S11 \| S6 AND S10 \| 2,342; S10 \| S7 OR S8 OR S9 \| 7,375; S9 \| TI ( (("area" or "brain" or "nucle*" or "lateral*" or "lucidum*" or "pelusidum*" or "region") N2 ("anterior commissure" or "diagonal band" or "septal*" or "septum*" or "stria*" or "supracommissural" or "terminali*")) ) OR AB ( (("area" or "brain" or "nucle*" or "lateral*" or "lucidum*" or "pelusidum*" or "region") N2 ("anterior commissure" or "diagonal band" or "septal*" or "septum*" or "stria*" or "supracommissural" or "terminali*")) ) \| 6,395; S8 \| TI "Paraterminal Bod*" OR AB "Paraterminal Bod*" \| 0; S7 \| DE "Septal Nuclei" \| 2,077; S6 \| S1 OR S2 OR S3 OR S4 OR S5 \| 290,637; S5 \| TI ( ((“brain*” or “neur*”) N2 (“activ*” or “connectiv*” or “correlate*” or “function*” or “image” or “images” or “imaging*” or “mapping*” or “network*”)) ) OR AB ( ((“brain*” or “neur*”) N2 (“activ*” or “connectiv*” or “correlate*” or “function*” or “image” or “images” or “imaging*” or “mapping*” or “network*”)) ) \| 188,884; S4 \| TI ( (“echoplanar*” or “echo-planar*” or “connectom*” or “functional cerebral localization*” or “fMRI” or “MRI” or “PET-MRI” or “PET” or “NMR” or “magnetoencephalogra*” or “magneto-encephalogra*” or “magneto encephalogra*” or “MEG” or “neuroimag*” or “zeugmatograph*”) ) OR AB ( (“echoplanar*” or “echo-planar*” or “connectom*” or “functional cerebral localization*” or “fMRI” or “MRI” or “PET-MRI” or “PET” or “NMR” or “magnetoencephalogra*” or “magneto-encephalogra*” or “magneto encephalogra*” or “MEG” or “neuroimag*” or “zeugmatograph*”) ) \| 109,263; S3 \| TI ( (“chemical shift*” or “functional” or “magnetic resonance” or “MR” or “magneti?ation transfer*” or “positron-emission*” or “proton spin*” or “spin echo*”) and (“image” or “images” or “imaging” or “scan” or “scans” or “tomograp*”) ) OR AB ( (“chemical shift*” or “functional” or “magnetic resonance” or “MR” or “magneti?ation transfer*” or “positron-emission*” or “proton spin*” or “spin echo*”) and (“image” or “images” or “imaging” or “scan” or “scans” or “tomograp*”) ) \| 74,316; S2 \| (((DE "Magnetic Resonance Imaging" OR DE "Diffusion Tensor Imaging" OR DE "Functional Magnetic Resonance Imaging") OR (DE "Magnetoencephalography")) OR (DE "Positron Emission Tomography")) OR (DE "Neural Networks") \| 113,149; S1 \| (DE "Neuroimaging") \| 22,311 | |
